# Comprehensive Analyses of Four *PhNF-YC* Genes from *Petunia hybrida* and Impacts on Flowering Time

**DOI:** 10.3390/plants13050742

**Published:** 2024-03-06

**Authors:** Jing Bin, Qinghua Tan, Shiyun Wen, Licheng Huang, Huimin Wang, Muhammad Imtiaz, Zhisheng Zhang, Herong Guo, Li Xie, Ruizhen Zeng, Qian Wei

**Affiliations:** 1 Guangdong Province Key Laboratory of Plant Molecular Breeding, College of Forestry and Landscape Architecture, South China Agricultural University, Guangzhou 510642, China; 20212159001@stu.scau.edu.cn (J.B.); 2022204026@stu.njau.edu.cn (S.W.); hlcheng@stu.scau.edu.cn (L.H.); zszhang@scau.edu.cn (Z.Z.); guoherong@scau.edu.cn (H.G.); xieli@scau.edu.cn (L.X.); zengrz@scau.edu.cn (R.Z.); 2 College of Horticulture, South China Agricultural University, Guangzhou 510642, China; 13113690728@stu.scau.edu.cn (Q.T.); huiminwang@stu.scau.edu.cn (H.W.); 3 College of Horticulture, Nanjing Agricultural University, Nanjing 210095, China; 4 Department of Horticulture, Abdul Wali Khan University, Mardan 23200, Pakistan; mimtiaz@awkum.edu.pk

**Keywords:** *PhNF-YC*, VIGS, flowering time, *Petunia hybrid*

## Abstract

Nuclear Factor Y (NF-Y) is a class of heterotrimeric transcription factors composed of three subunits: NF-A, NF-YB, and NF-YC. NF-YC family members play crucial roles in various developmental processes, particularly in the regulation of flowering time. However, their functions in petunia remain poorly understood. In this study, we isolated four *PhNF-YC* genes from petunia and confirmed their subcellular localization in both the nucleus and cytoplasm. We analyzed the transcript abundance of all four *PhNF-YC* genes and found that *PhNF-YC2* and *PhNF-YC4* were highly expressed in apical buds and leaves, with their transcript levels decreasing before flower bud differentiation. Silencing *PhNF-YC2* using VIGS resulted in a delayed flowering time and reduced chlorophyll content, while *PhNF-YC4*-silenced plants only exhibited a delayed flowering time. Furthermore, we detected the transcript abundance of flowering-related genes involved in different signaling pathways and found that *PhCO*, *PhGI*, *PhFBP21*, *PhGA20ox4*, and *PhSPL9b* were regulated by both *PhNF-YC2* and *PhNF-YC4*. Additionally, the transcript abundance of *PhSPL2*, *PhSPL3*, and *PhSPL4* increased only in *PhNF-YC2*-silenced plants. Overall, these results provide evidence that *PhNF-YC2* and *PhNF-YC4* negatively regulate flowering time in petunia by modulating a series of flowering-related genes.

## 1. Introduction

Flowering at the proper time is essential for reproductive success in plants. To ensure timely blooming, plants have developed various pathways that respond to internal signals and environmental cues. These pathways involve the convergence of external stimuli, such as photoperiod and temperature, as well as internal conditions like plant age and levels of gibberellic acid. Together, these factors regulate a set of floral integrator genes, such as *FLOWERING LOCUS T* (*FT*), *LEAFY* (*LFY*), *SUPPRESSOR OF OVEREXPRESSION OF CO1* (*SOC1*), and *APETALA 1* (*AP1*), which transmit floral inductive or repressive signals to the shoot apical meristem (SAM) [1,2]. To date, many key genes in different flowering pathways have been uncovered. For instance, *CONSTANS* (*CO*) plays a crucial role in the photoperiod pathway, while *VERNALIZATION1* (*VRN1*), *VRN2*, and *FLOWERING LOCUS C* (*FLC*) are essential in the vernalization pathway. In the gibberellic acid (GA) pathway, *GA3-oxidase* (*GA3ox*), and *GA20-oxidase* (*GA20ox*) are key enzyme genes, while *SQUAMOSA PROMOTER BINDING PROTEIN-LIKE* (*SPL*) genes are key components in the aging pathway.

Nuclear factor Y (NF-Y) transcript factors have been reported to be involved in many processes, such as flowering time [3,4,5,6,7], chloroplast biogenesis [8], root growth [9], and stress response [10,11,12,13,14,15,16]. NF-Y is a heterotrimeric transcription factor that specifically binds to the CCAAT box in the promoter regions of many genes in all eukaryotes [17]. The NF-Y complex comprises three subunits: NF-YA, NF-YB, and NF-YC. All three NF-Y subunits are necessary for the formation and transcriptional activity of the complex [18,19], and they are encoded by multigene families in plants [20,21]. In *Arabidopsis*, 10 NF-YAs, 13 NF-YBs, and 13 NF-YCs have been identified, resulting in the theoretical possibility of 1690 unique heterotrimeric complexes that can specifically regulate numerous downstream genes [22].

NF-Y members, especially the NF-YB and NF-YC family members, have been found to play a role in the regulation of flowering time. In *Arabidopsis*, the individual *NF-YBs* (*AtNF-YB2* and *AtNF-YB3*) and *NF-YCs* (*AtNF-YC3*, *AtNF-YC4*, and *AtNF-YC9*) promote flowering by inducing the expression of *AtFT* under long-day (LD) conditions [5,7]. Subsequent studies demonstrated that AtNF-YC proteins (AtNF-YC3, AtNF-YC4, and AtNF-YC9) physically interact with AtNF-YB2 and AtNF-YB3 to form heterodimer complexes to regulate the timing of flowering [23,24]. Additionally, these NF-YC proteins can also interact with ABA-responsive element-binding factors (ABFs) to promote flowering by increasing the transcription of *AtSOC1* under drought conditions [25]. The overexpression of *AtNF-YC2* results in early flowering, which is associated with increased *AtFT* transcripts [3]. The involvement of NF-Y subunits in flowering time regulation is conserved in other plant species as well. In rice, *OsNF-YC2* and *OsNF-YC4* inhibit flowering, while *OsNF-YC6* promotes flowering by modulating the expression of *Early heading date 1* (*Ehd1*), *Heading date 3a* (*Hd3a*), and *RICE FLOWERING LOCUS T1* (*RFT1*) under LD conditions [4,26]. Moreover, it has been proven that OsNF-YC2/4/6 proteins physically interact with OsNF-YB8/10/11 proteins in vitro [26]. In wheat, NF-Y proteins interact with the CCT domain of Vernalization2 (VRN2) and CONSTANTS2 (CO2) to play a role in integrating the vernalization and photoperiod signals, which are major environmental cues for flowering in plants [27]. In chrysanthemums, CmNF-YB8 delays flowering time by directly regulating *miR156*, an essential factor in the aging pathway [6]. Additionally, overexpressing *BdNF-YB3* and *BdNF-YB6* recuses the late flowering phenotypes of an *Arabidopsis nf-yb2nf-yb3* double mutant [21].

Multiple studies have shown that NF-Y proteins play roles in chloroplast biogenesis and photomorphogenesis. In wheat, *TaNF-YC11* is co-regulated with a series of photosynthesis-related genes such as *TaLHCII*, *TaCAB*, and *TaLHCI* [28]. In rice, the silencing of *OsHAP3A* (a member of NF-YB subunits) leads to pale green leaves, accompanied by a reduced chlorophyll content and degenerated chloroplasts [8]. The overexpression of *ZmNF-YB2* in maize enhances drought tolerance by increasing the chlorophyll content and the stomatal conductance and photosynthesis rates [11]. In *Arabidopsis*, *NF-YA5* and *NF-YB9* have been found to be involved in the regulation of light-harvesting chlorophyll a/b-binding protein [29].

In petunia (*Petunia hybrida*), the study of flowering pathways has been limited, but some flowering-related genes have been identified. The MADs family, including *PETUNIA FLOWERING GENE (PFG)*, *FLORAL BINDING PROTEIN26 (FBP21)*, *FBP20 (UNSHAVEN)*, and *PhFT*, have been found to be involved in the regulation of flowering time [30,31,32,33,34,35]. The circadian clock gene *GIGANTEA* (*GI*), a component in photoperiod pathway, positively regulates flower initiation and flower maturation [36]. Multiple *SPL* genes, *SBP1*, *SPB2*, *SPL9a*, and *SPL9b*, positively regulate flowering time in petunia [37,38]. Moreover, *CONSTANS-LIKE16* (*COL16*) has been reported to positively regulate chlorophyll content [39]. However, the functions of *NF-YC* genes in petunia have not been extensively studied. In this study, an expression analysis, subcellular localization, virus-induced gene silencing (VIGS), and a phenotype examination were conducted. Among the four *phNF-YCs*, *PhNF-YC2* and *PhNF-YC4* are involved in flowering time regulation. The expression patterns of flowering-related genes were also examined in VIGS-mediated *PhNF-YC2*-silenced plants and *PhNF-YC4*-silenced plants, which supports the conclusion that these NF-YC genes negatively regulate flowering time through multiple pathways.

## 2. Results

### 2.1. Cloning and Sequence Analysis of PhNF-YCs

**Figure 1:**
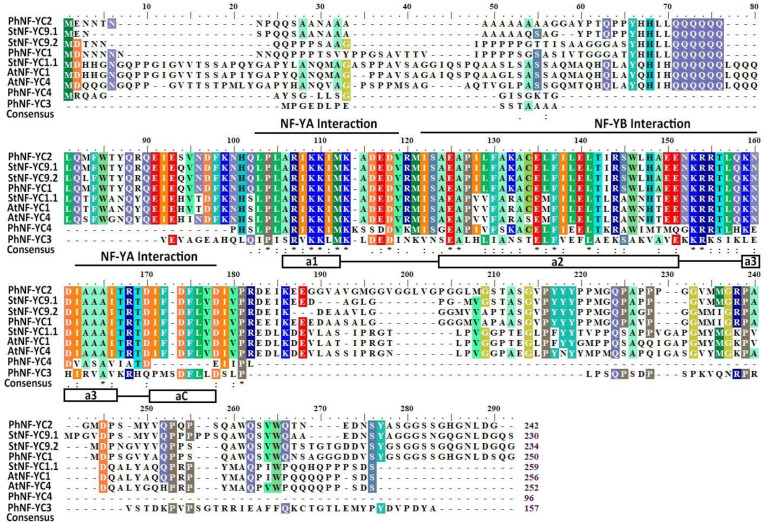
Multiple sequence alignment of PhNF-YC proteins with NF-YC proteins from other plant species. The DNA-binding and subunit interaction domains are shown above the alignment. The secondary structures, alpha helices, and strand loops are represented underneath the alignment. * represents completely conserved amino acids in different species. Ph, *Petunia hybrida*; At, *Arabidopsis thaliana*; St, *Solanum tuberosum*.

According to the draft genome sequence of *Petunia axillaris*, four NF-YC family members were identified. To obtain the corresponding *NF-YC* genes of *Petunia hybrida* ‘Jimei’, the cDNA of *P. hybrida* ‘jimei’ was used as a template for PCR amplification, which resulted in four fragments ranging from 977 bp to 1163 bp in length, with open reading frame (ORF) regions ranging from 327 bp to 780 bp (Table S1). All four PhNF-YC proteins were found to have a core histone sequence consisting of four alpha helices separated by three strand-loop domains, indicating that they belong to the PhNF-YC transcription factor family (Figure 1).

**Figure 2:**
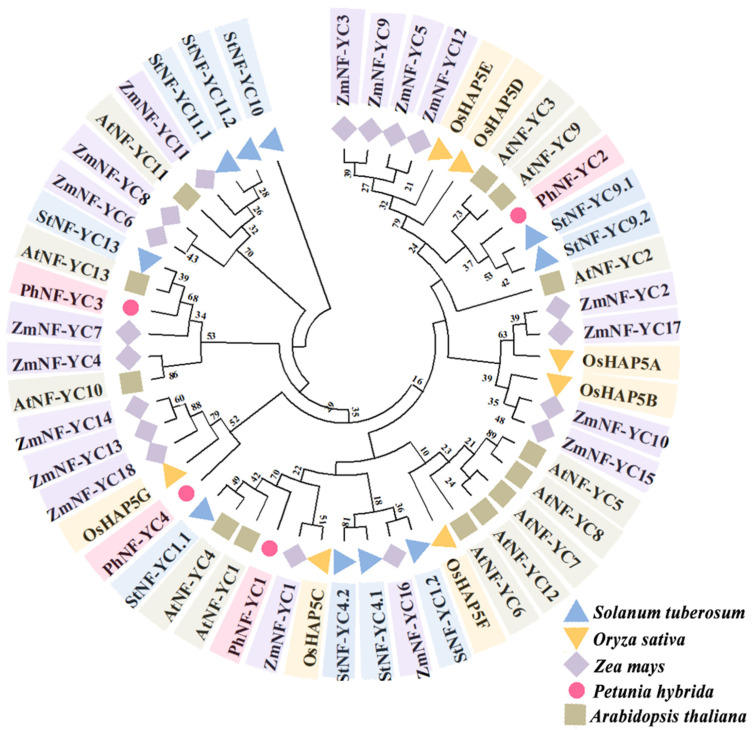
A phylogenetic analysis of PhNF-YC proteins and NF-YC proteins from other plant species. The values on the branches indicates the probability and the likelihood of obtaining this result in 1000 repetitions. Ph, *Petunia hybrida*; At, *Arabidopsis thaliana;* St, *Solanum tuberosum;* Zm, *Zea mays;* Os, *Oryza sativa*.

To further analyze the genetic relationship between the PhNF-YC proteins and NF-YC proteins in other species, a comprehensive phylogenetic tree was constructed using NF-YC proteins from a variety of monocot and dicot plants (Table S2). The results showed that NF-YCs were conserved throughout evolution, and PhNF-YCs were distributed in different branches. This suggested that PhNF-YCs might have different functions. PhNF-YC1, AtNF-YC1, AtNF-YC4, and StNF-YC1.1 were clustered in the same branch, while PhNF-YC2 was clustered in the branch of AtNF-YC3 and AtNF-YC9. PhNF-YC3 showed the closest relationship with AtNF-YC13, StNF-YC13, ZmNF-YC3, ZmNF-YC7, and AtNF-YC10, while PhNF-YC4 had the closest relationship with ZmNF-YC13, ZmNF-YC14, ZmNF-YC18, and OsHAP5G. The clustering results reveal a decline in the number of NF-YC family members in petunia over the course of evolution, which should be accompanied by a corresponding decrease in functional redundancy within the family (Figure 2).

### 2.2. Transcript Profiles of PhNF-YC Genes

**Figure 3:**
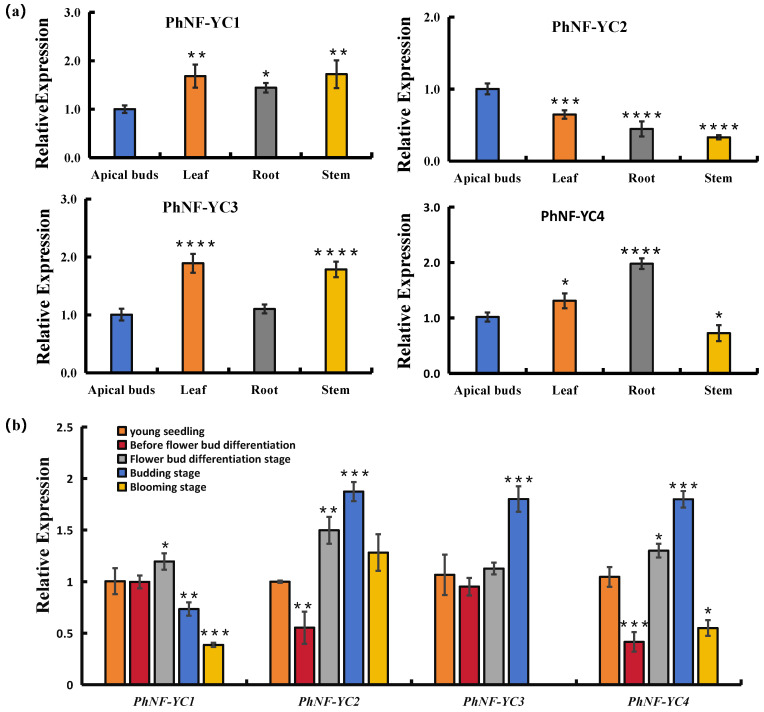
Transcript abundances of petunia *PhNF-YC* genes in different organs and developmental stages. (**a**) Transcript abundances of *PhNF-YCs* in different organs. *, **, ***, and **** indicate significant differences from the relative expression of apical buds at *p*-values < 0.05, 0.01, 0.001, and 0.0001, respectively. (**b**) Transcript abundances of *PhNF-YCs* in different developmental stages. *, **, and *** indicate significant differences from the relative expression of young seedlings at *p*-values < 0.05, 0.01, and 0.001, respectively.

To understand the biological functions of *PhNF-YCs* in petunia, their expression patterns were analyzed in different organs of petunia plants without flower bud differentiation. As shown in Figure 3a, the transcript abundance of *PhNF-YC1* was found to be the lowest in apical buds compared to other organs. *PhNF-YC2* exhibited the highest transcript abundance in apical buds, followed by leaves. *PhNF-YC3* had a relatively higher transcript abundance in leaves and stems, while *PhNF-YC4* exhibited the highest expression levels in roots, followed by leaves and apical buds. 

To further investigate the roles of *PhNF-YCs* in flowering, their transcript abundance was evaluated at different developmental stages (Figure 3b). The abundance of *PhNF-YC1* showed no significant difference before flower bud differentiation and then gradually decreased with development. The transcript level of *PhNF-YC3* was the highest during the budding stage and was not detected during the blooming stage. Both *PhNF-YC2* and *PhNF-YC4* exhibited a sharp decrease before flower bud differentiation followed by a rapid increase, reaching about two-fold of the levels of young seedlings and finally decreased in the blooming stage. These results suggest that the decrease in the expression of *PhNF-YC2* and *PhNF-YC4* before flower bud differentiation may be related to flower induction, while *PhNF-YC3* may play a role in flower blooming.

### 2.3. Subcellular Localization of PhNF-YC Proteins

**Figure 4:**
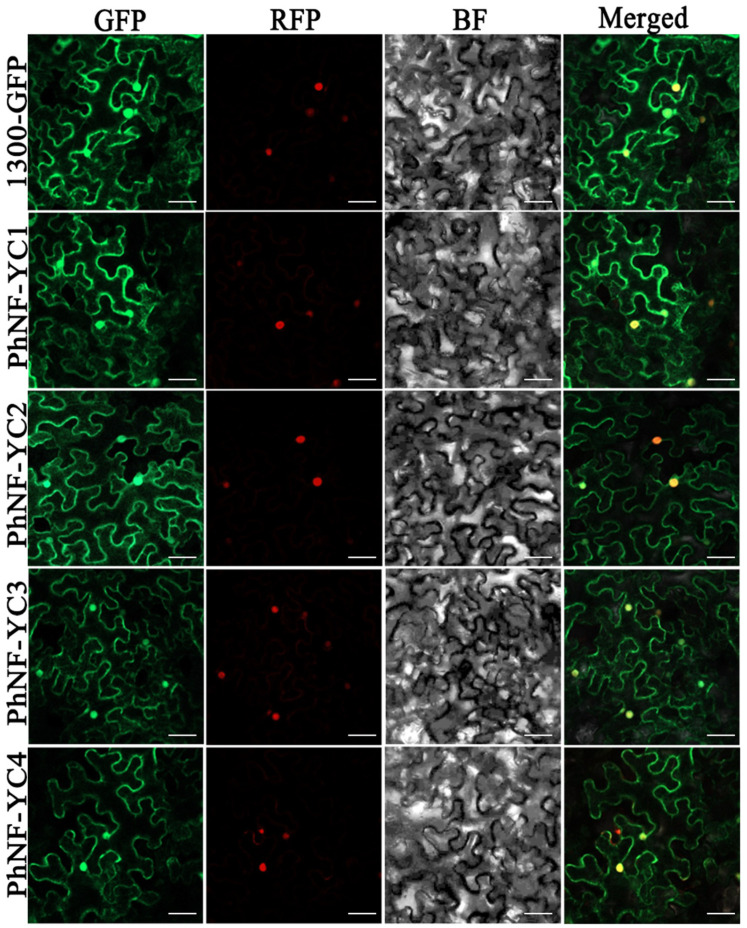
Transient expression of PhNF-YCs-GFP fusion protein in *N. benthamiana* leaves. Fluorescent signals were visualized by confocal microscopy 72 h after infiltration. H2B-mCherry was used as a marker to indicate nuclei. Scale bars = 50 μm.

To investigate the subcellular localization of the PhNF-YC proteins, a fusion protein comprising PhNF-YCs fused to a green fluorescent protein (PhNF-YCs-GFP) driven by the 35S promoter was introduced into tobacco leaves for transient expression. The results revealed that all PhNF-YCs-GFP fusion proteins were observed in both the cytoplasm and the nuclei of the cells (Figure 4). 

### 2.4. Silencing PhNF-YCs Influences the Flowering Time of Petunia

**Figure 5:**
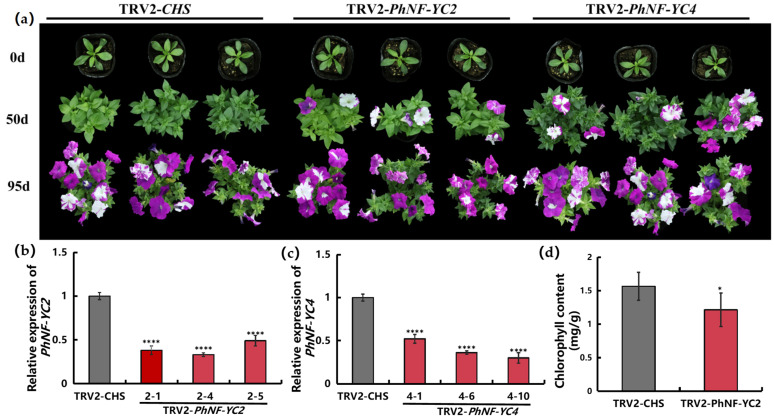
Phenotypical alteration of VIGS-mediated silencing of *PhNF-YC2* and *PhNF-YC4* in petunia. (**a**) Phenotypes of VIGS-mediated *PhNF-YC2*-silenced plants and *PhNF-YC4*-silenced plants at 50 days and 95 days after infection; (**b**) relative expression levels of *PhNF-YC2* in control plants and *PhNF-YC2*-silenced plants; (**c**) relative expression levels of *PhNF-YC4* in control plants and *PhNF-YC4*-silenced plants; (**d**) average total chlorophyll content of control plants and *PhNF-YC2*-silenced plants. Three independent experiments were performed, and error bars indicate standard deviation. * and **** indicate significant differences from the relative expression or chlorophyll content of control plants (TRV-*CHS*) at *p*-values < 0.05 and 0.0001, respectively.

To investigate whether *PhNF-YCs* influence flowering time, the untranslated regions (UTRs) of PhNF-YCs were utilized to specifically silence the expression of individual *PhNF-YC* genes in petunia through VIGS. Three weeks after infection, the transcript levels of the *PhNF-YCs* decreased by 40–70% in the plants infected with the TRV2-*PhNF-YC* constructs compared to the control plants infected with the TRV2-*CHS* construct (Figure 5b,c). Except for the target gene, the transcript abundance values of the other *PhNF-YC* genes showed no differences between the plants infected with the TRV2-*PhNF-YC* construct and the control plants (Figure S1). Compared to the control plants, there were no notable phenotypic differences observed in the *PhNF-YC1*-silenced plants or the *PhNF-YC3*-silenced plants. However, compared to the control plants, both the *PhNF-YC2*-silenced plants and *PhNF-YC4*-silenced plants exhibited significantly earlier flowering at 50 days after infection, and these differences disappeared once the plants entered the blooming stage (Figure 5a). At 50 days after infection, bloomed flowers were observed in approximately 90% of *PhNF-YC2*-silenced plants, while only 12% of the control plants had flower buds. Additionally, the *PhNF-YC2*-silenced plants showed a lighter leaf color, and the total chlorophyll content was measured to be 0.99 mg·g^−1^, whereas the control plants had a chlorophyll content of 1.56 mg·g^−1^ (Figure 5d). 

### 2.5. Silencing PhNF-YCs Changes the Expression of Flowering-Related Genes

To gain insights into the mechanism through which PhNF-YC2 and PhNF-YC4 regulate flowering time, the transcript levels of various flowering-related genes were quantified in petunia using an RT-qPCR. *PhGI* and *PhCO* are key genes in the photoperiod pathway; *GA20ox2*, *3*, and *4* play crucial roles in the GA biosynthesis pathway. *PhSPLs* are key genes involved in the aging pathway, and *PhFBP21* (the homolog of *AtSOC1*) is a common downstream integrator in the flowering regulatory network.

**Figure 6:**
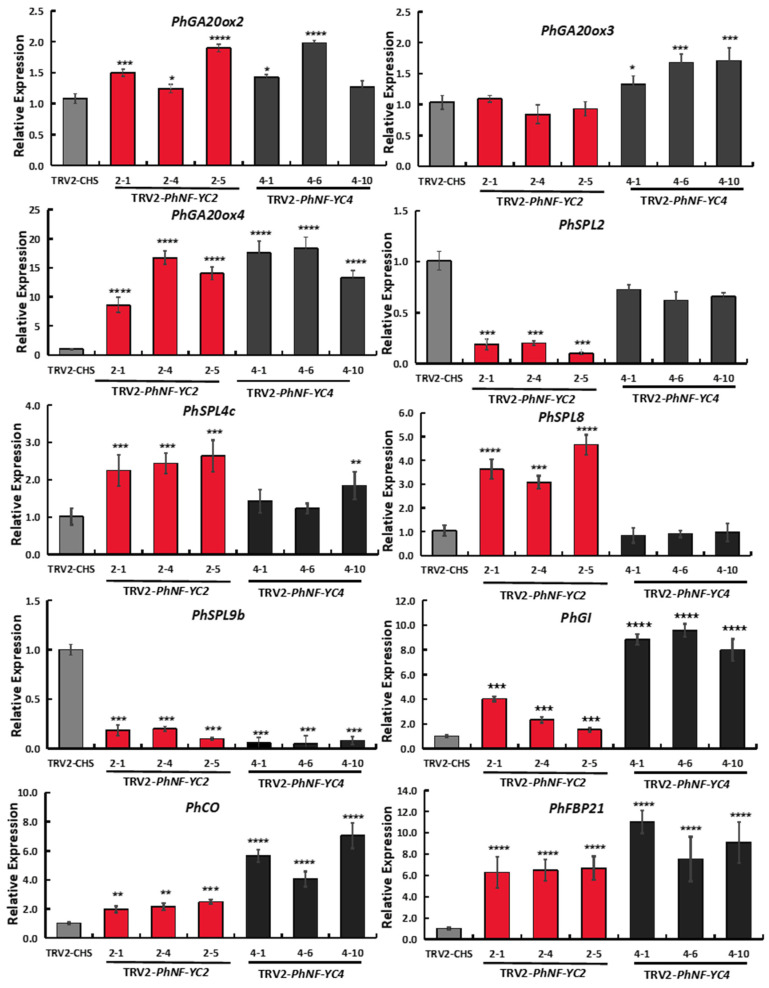
Transcript abundances of flowering-related genes in *PhNF-YC2-silenced* and *PhNF-YC4-silenced* plants. Three independent experiments were performed, and error bars indicate standard deviation values. *, **, *** and **** indicate significant differences from the relative expression of control plants (TRV-*CHS*) at *p*-values < 0.05, 0.01, 0.001, and 0.0001, respectively.

The results showed that the transcript levels of *PhGA20ox4*, *PhCO*, *PhGI*, and *PhFBP21* were significantly increased in both the *PhNF-YC2*-silenced plants and *PhNF-YC4-silenced* plants, while the transcript level of *PhSPL9b* was decreased (Figure 6). Moreover, the expression levels of *PhSPL2, PhSPL4, and PhSPL8* were significantly changed in *PhNF-YC2*-silenced plants alone, while *PhGA20ox3* was only increased in *PhNF-YC4-silenced* plants. These results suggest that *PhNF-YC2* and *PhNF-YC4* jointly regulate certain flowering genes; however, *PhNF-YC2* plays a more critical role in the aging pathway, while *PhNF-YC4* has a distinct function in the GA pathway.

## 3. Discussion

The regulation of flowering time in plants has been extensively studied, particularly using *Arabidopsis thaliana* as a model system. Previous research revealed that the timing of flowering is controlled by a complex regulatory network that integrates environmental cues, such as photoperiod and temperature, with internal factors like age and hormone signaling, specifically gibberellins [2,40]. Many genes involved in these pathways and their interactions have been identified. However, recent studies have highlighted significant crosstalk between certain transcription factor families and these well-defined pathways [41,42]. Some *NF-YC* family genes have been shown to regulate flowering time through photoperiod-dependent pathway in *Arabidopsis*, rice, and tobacco [3,4,5,16,28]. In petunia, there are 27 members of the NF-Y family, including 10 PhNF-YAs, 13 PhNF-YBs, and 4 PhNF-YCs [43]. Until now, there were no reports on the functions of PhNF-YCs. We attempted to identify the function of NF-YC in *Petunia hybrida* ‘Ji mei’. According to the PlantTFDB database, there are 11 NF-YC family members in the reported petunia genome (Table S3). However, upon conducting an analysis, it was observed that five of these members, namely Peaxi162Scf00050g02112, Peaxi162Scf00686 g00043, Peaxi162scf00877g00127, Peaxi162scf00978g00427, and Peaxi162scf 47032g00004, do not possess typical NF-YC domains. As a result, these five members were not subjected to PCR amplification in *Petunia hybrida* ‘Ji mei’. The sequences of the remaining six members were successfully obtained through PCR amplification using high-fidelity enzymes. However, it was found that both Peaxi162Scf00220g00230 and Peaxi162scf0076 3a00212 lack one NF-YA-binding domain, which hinders the formation of an α helix structure. Consequently, these two proteins were excluded from further studies. Finally, the remaining four NF-YC members were selected for our study.

To investigate whether PhNF-YCs are involved in the regulation of flowering time, the transcript levels of four *PhNF-YCs* were analyzed in different tissues and developmental stages. Previous studies have shown that most flowering-time genes are mainly expressed in the leaves, shoot apex, and meristem. For tissue expression detection, we selected 50-day-old petunia seedlings, in which flower bud differentiation occurs two weeks later, to collect tissue samples. We focused on members that showed high levels of expression in both apical buds and leaves. *PhNF-YC1* was highly expressed in stem and leaf tissues. Moreover, its expression levels did not differ from the seedling stage to the budding stage, suggesting that *PhNF-YC1* may be involved in developmental processes other than flowering time regulation (Figure 3a,b). *PhNF-YC3* was highly expressed in leaves, with the lowest level of expression in apical buds. At the same time, *PhNF-YC3* showed a relatively lower level of expression compared to other members, indicating that it may not be a key gene in flowering progress. Notably, *PhNF-YC2* and *PhNF-YC4* exhibited a sharp decrease before flower bud differentiation, indicating possible negative roles in flowering; and the increased expression levels of *PhNF-YC2*, *3* and *4* during the budding stage, suggesting that these three members might exhibit functional redundancy in flower blooming. All *PhNF-YCs* exhibited a decreasing trend in expression levels after the budding stage, suggesting that they may play negative or limited roles in flowering senescence.

In *Arabidopsis*, *NF-YC3*, *NF-YC4* and *NF-YC9* have been reported to positively regulate flowering time under LD conditions in a functionally redundant manner [5]. This functional redundancy may be due to their highly similar amino acid sequences. On the other hand, *OsNF-YC2* and *OsNF-YC4*, the homologs of *AtNF-YC3/4/9*, play negative roles in regulating flowering time under LD conditions [4]. In petunia, only four NF-YC members were identified, and PhNF-YC2 was the only one close to AtNF-YC3 and AtNF-YC9 (Figure 2), suggesting that functional redundancy may not exist. In our study, the silencing of *PhNF-YC2* resulted in early flowering, which was consistent with the function of *OsNF-YC2* and *OsNF-YC4*. These results indicate that homologous genes of NF-YC family members in different species may have similar or opposite functions. PhNF-YC4 was found to have the closest relationship with ZmNF-YC13, ZmNF-YC14, ZmNF-YC18, and OsHAP5G (Figure 2), none of which have been reported to regulate flowering time. Therefore, the function and mechanism of PhNF-YC4 in regulating flowering time are both worth further exploration and verification.

In our study, we observed that the silencing of *PhNF-YC2* not only resulted in early flowering but also a less obvious smaller crown diameter (not mentioned in the text) and lighter leaf color (Figure 5a,d), which was not mentioned in previous NF-YC related studies. However, a similar phenomenon has been reported in research on the NF-YB subfamily. The ectopic overexpression of *PdNF-YB7* in *Arabidopsis* resulted in an increased leaf area, while a *nf-yb7* mutant showed a decreased leaf area [12]. The overexpression of *CmNF-YB8* in *Arabidopsis* reduced crown diameter, which was due to changes in the expression of genes involved in the aging pathway [6]. Furthermore, *NF-YB* genes positively regulated the chlorophyll content in rice and maize. It is known that three subunits of NF-Y usually form a heterotrimer which then functions as a transcription factor. In petunia, there were only four NF-YC members, a far fewer number than in other plants; for example, there are 10 members in *Arabidopsis*, 15 members in Soybean [44], and 12 members in Brachypodium [21]. The fewer members in the PhNF-YC family may result in a reduction in heterotrimers, leading to a decrease in the functional redundancy of NF-Y in petunia. The silencing of *PhNF-YC2* may result in a lack of heterotrimer complexes related to the crown diameter phenotype. Thus, both the functions of NF-YC and the NF-Y heterotrimer need to be further explored.

Our study revealed that the reduction in *PhNF-YC2* and *PhNF-YC4* expression induced by viruses played a critical role in promoting early flowering. As viruses can move within infected plants, *PhNF-YCs* likely had multiple mechanisms involved in regulating the flowering process. For instance, they might directly modulate the expression of genes, such as *FT*, in meristematic tissues to initiate flowering. Previous research studies demonstrated that NF-YC formed heterotrimers with NF-YB and NF-YA to bind to CCAAT elements in promoter sequences and regulate downstream gene expression [18,19]. Many NF-Y members were involved in various flowering pathways, including the photoperiod pathway [4,5,26], vernalization pathway [27], GA pathway [45], and aging pathway [6]. Due to the fact that signals from different flowering pathways ultimately converge on downstream integrators such as *FT*, *LFY*, and *SOC1*, changes in the expression levels of integrator genes can assist in confirming changes in the flowering phenotype and are not sufficient to determine which specific flowering pathway is affected by NF-YC. Therefore, our research focused on studying the specific pathways through which NF-YC influences flowering time, closely monitoring changes in gene expression levels in each pathway.

The expression levels of reported flowering-related genes in petunia were detected. The expression of *PhSPL9b* was decreased in *PhNF-YC2*-silenced plants and *PhNF-YC4*-silenced plants (Figure 6). However, *PhSPL9b* was reported to be a positive regulator of flowering time [38]. In *Arabidopsis*, the homolog of *PhSPL9*, AtSPL9, has been shown to have a positive role in maintaining juvenile growth. Therefore, the observed decrease in *PhSPL9b* expression in our study could potentially be attributed to the specific sampling stage or growth stages, which can influence gene expression levels. Further investigation is necessary to comprehensively comprehend the role of *PhSPL9b* in regulating flowering time in petunia. In our study, the transcript levels of key genes involved in the photoperiod pathway and age pathway were significantly changed in *PhNF-YC2*-silenced plants (Figure 6), but whether *PhNF-YC2* regulated flowering time through the aging pathway or photoperiod pathway still needs further study. In previous studies, it was demonstrated that NF-Y regulateds flowering time through multiple flowering pathways, which was consistent with our result. The situation was the same for *PhNF-YC4*. To further confirm the functions of *PhNF-YC2* and *PhNF-YC4*, transgenic methods could be employed. Additionally, exploring the underlying mechanisms of their action would be valuable for gaining a deeper understanding of their roles in flowering time regulation.

## 4. Materials and Methods

### 4.1. Plant Materials 

*Petunia hybrida* ‘Jimei’ seeds were planted in a 96-well plastic tray filled with a mixture of 1:1 (*v*/*v*) peat and pearlite and germinated in a culture room at 22–25 °C with a 14 h light/10 h dark cycle. After approximately 30 days, when the seedlings reached the 4-leaf stage, they were transferred to 9 cm diameter pots containing a mixture of 1:1 (*v*/*v*) peat and pearlite. At the 7–8-leaf stage, the seedlings were subjected to infection using Agrobacterium tumefaciens strain GV3101 carrying either TRV2-*CHS* or TRV2-*PhNF- YCs* constructs.

### 4.2. Isolation of Petunia PhNF-YC Gene

Full-length *PhNF-YC* sequences were obtained from the SGN database (https://www.solgenomics.net/, accessed on 6 June 2022) and used as reference sequences for primer design. The total RNA was extracted from the mature leaves of ‘Jimei’ using Trizol reagent (TaKaRa, Kyoto, Japan) according to the manufacturer’s instructions. The UTR and coding sequences of the PhNF-YCs were then obtained by an RT-PCR using gene-specific primers (Table S4). The PCR products were purified and cloned into a pGEM-T Easy Vector (Promega, Madison, WI, USA) for sequencing.

### 4.3. Phylogenetic Analysis

Alignments of the PhNF-YC proteins with NF-YC proteins from other species were performed using the ClustalW 2.0 program (http://www.genome.jp/tools/clustalw/, accessed on 6 June 2022) and BioEdit v7.2.6.1 (http://www.mbio.ncsu.edu/BioEdit/bioedit.html, accessed on 6 June 2022). A phylogenetic analysis was conducted using MEGA 6 software to compare the PhNF-YC proteins with all reported NF-YCs in *Arabidopsis thaliana*, *Solanum tuberosum*, *Zea mays*, and *Oryza sativa* [46,47,48,49]. A neighbor-joining tree was constructed using MEGA6 with the following parameters: the Jones–Taylor–Thorton model (JTT), gamma distributed (G), complete deletion, and 1000 bootstrap replicates [50].The displayed neighbor-joining tree represented a consensus bootstrap tree. The NF-YC proteins from other plant species were obtained from publicly available databases [47,48,49,50], including TAIR (https://www.arabidopsis.org/, accessed on 6 June 2022), the Rice Genome Research Program (http://rgp.dna.aVrc.go.jp/, accessed on 6 June 2022), the maize sequencing database (http://ensembl.gramene.org, accessed on 6 June 2022), and the SNG database (https://www.solgenomics.net, accessed on 6 June 2022).

### 4.4. Subcellular Localization of PhNF-YC Protein

The full-length coding sequences, except for the stop codons of PhNF-YCs, were amplified using primers containing 15–25 bp vector sequences (Table S4). The PCR products were introduced into the pCAMBIA 1300-GFP vector between *Xba* I and *Spel* using a *Trelief* ^TM^ sosoo cloning kit (TsingKE, Beijing, China). The p2300-35s-H2B-mCherry vector was used as a reference for nuclear localization. Constructs were transformed into Agrobacterium strain EHA105 and subsequently infiltrated into the leaf epidermal cells of 3-week-old *Nicotiana benthamiana*. The plants were incubated at 25 °C for 72 h in the dark, and the cells were then observed using a confocal laser scanning microscope (fv1000, Olympus, Shinjuku City, Japan).

### 4.5. Virus-Induced Gene Silencing

To generate TRV2 constructs containing the untranslated regions (UTRs) of *PhNF-YCs*, the UTR sequences were amplified via a PCR using specific primers containing 15–25 bp vector sequences (Table S4). The PCR products were cloned into a TRV2 vector that was digested with *EcoR* I and *BamH* I using a *Trelief* ^TM^ sosoo cloning kit (TsingKE, Beijing, China). The resulting TRV2-*PhNF-YCs* plasmids were introduced into *Agrobacterium tumefaciens* strain GV3101. *A. tumefaciens* containing TRV1 and TRV2 derivatives were prepared as previously described [51]. The agrobacterium cells grown overnight were harvested and resuspended in inoculation buffer (10 mM of MES, 200 μM of acetosyringone, and 10 mM of MgCl_2_) to an OD600 of 2.0. After a 3–5 h of incubation at room temperature, bacteria bearing TRV1 and TRV2 or TRV2-*PhNF-YC2* were mixed in equal volumes, and 1 mL of this mixture was injected into the leaves of petunia plantlets. Twenty plants were infiltrated for each silencing target. The plants were then moved into a culture room maintained at 22–25 °C with a 14 h light/10 h dark cycle. 

### 4.6. Chlorophyll Extraction and Analysis

Ten *PhNF-YC2*-silenced plants and ten control plants were used to measure the chlorophyll content. The second fully expanded leaves from the tops of the plants were sampled. For every sample, 0.2 g of leaf tissue was ground in liquid nitrogen. The samples were incubated overnight in 80% acetone to extract chlorophyll and then centrifuged at 7000 rpm for 10 min. The value of absorbance was measured at 665 nm and 649 nm using a spectrophotometer (RS232C, Eppendorf, Hamburg, Germany). The concentration of chlorophyll a and b and total were calculated according to the Lichtenthaler method [52].

### 4.7. Quantitative Real-Time PCR Assays

To assess the tissue-specific transcript profiles of *PhNF-YCs*, samples of apical buds, leaves, stems, and roots were harvested from 50-day-old petunia seedlings. To evaluate the transcript profiles of the *PhNF-YCs* at different developmental stages, the apical buds of the plants were sampled at the seedling stage (40 d), before the flower bud differentiation stage (55 d), during the flower bud differentiation stage (75 d), at the budding stage (105 d), and at the blooming stage (130 d). To detect the transcript abundance of flowering-related genes, the leaves were collected from the plants infected with the TRV2-*PhNF-YC2* construct and the TRV2-*PhNF-YC4* construct and the control plants on the 21st day after infection. Each sample had three biological replicates, and the collected samples were immediately frozen in liquid nitrogen and stored at −80 °C. 

RNA extraction was performed using the RNA Aprep Pure Plant Kit (Vazyme, Nanjing, China), following the manufacturer’s instructions. cDNAs were synthesized from 1 μg of total RNA utilizing a HiScript^®^ III 1st Strand cDNA Synthesis Kit (+gDNA wiper) (Vazyme, Nanjing, China). Quantitative real-time PCR reactions were performed using a CFX96 Real-Time PCR Detection System (Bio-Rad, Hercules, CA, USA) in standard mode, employing the ChamQ Universal SYBR qPCR Master Mix Kit (Vazyme, Nanjing, China). Gene-specific primers were listed in Table S1, and the petunia *CYP* gene (SNG number Peaxi162Scf00362g00960) was used as an internal control. Relative transcript abundances were normalized to the reference *PhCYP* gene via the 2 ^−∆∆Ct^ method [53]. Three independent experiments were conducted. The accession numbers of the flowering-related genes in this study are as follows: *PhCO:* SNG number Peaxi162Scf00047g01927; *PhGI:* SNG number Peaxi 132Scf1428Ctg026; *PhFBP21*: GenBank number AF335239; *PhSBP1*: GenBank number KT 717963; *PhSBP2*: GenBank number KT717964; *PhSPL2:* SNG number Peaxi162Scf00 128g01331; *PhSPL4c:* SNG number Peaxi162Scf00069g01624; *PhSPL8:* SNG number Peaxi 162Scf00001g00542; *PhSPL9b:* SNG number *Peaxi162Scf00003g04342; PhGA20ox1*: SNG numberPeaxi162Scf00988g00019; *PhGA20ox2*: SNG number Peaxi162Scf00132g00116; *PhGA20ox3*: Peaxi162Scf00000g00426; *PhGA20ox4*: Peaxi162Scf01178g00015.

The data were presented as mean ± standard error of the mean values. A statistical analysis was performed using GraphPad Prism version 8 (GraphPad Software Inc., San Diego, CA, USA). The error bars on the graphs were calculated based on a one-Way ANOVA. The following symbols were used to indicate statistically significant differences: for *p* < 0.05, “*”; for *p* < 0.01, “**”; for *p* < 0.001, “***”; and for *p* < 0.0001, “****”.

## 5. Conclusions

In this study, four *PhNF-YC* family genes were isolated and characterized from petunia. The expression profiles of the *PhNF-YC* genes across different tissues and developmental stages were assessed using an RT-qPCR. Furthermore, this study demonstrated the negative impact of *PhNF-YC2* and *PhNF-YC4* on flowering regulation through VIGS. This research study also explored regulatory pathways involving *PhNF-YC2* and *PhNF-YC4* by evaluating the expression levels of key genes in various flowering pathways. *PhNF-YC2* was found to modulate flowering time through the photoperiod, gibberellic acid (GA), and aging pathways, either directly or indirectly, while *PhNF-YC4* played a crucial role in the photoperiod and GA pathways. These findings provide a foundation for future investigations into the functions and regulatory mechanisms of PhNF-YCs in petunia.

## Data Availability

Data will be made available on request.

## Supplementary Materials

The following supporting information can be downloaded at https://www.mdpi.com/article/10.3390/plants13050742/s1, Figure S1: Relative expression levels of other *PhNF-YCs* in the *PhNF-YC2*-silenced plants and *PhNF-YC4*-silenced plants; Table S1: The nucleotide sequences of PhNF-YCs; Table S2: The amino acid sequences of NF-YCs in different species; Table S3: Summary of the characteristics of PhNF-YC members in PlantTFDB; Table S4: Primers used for this study.
