# Supplementary material for: Comprehensive Analyses of Four PhNF-YC Genes from Petunia hybrida and Impacts on Flowering Time
Source: Plants (Basel). 2024 Mar 6;13(5):742. doi: 10.3390/plants13050742 (PMC10934205; doi:10.3390/plants13050742)
Supplement: Supplementary file 1 [file plants-13-00742-s001.zip › Figure S1 Relative expression levels of flowering related genes in the control plants and VIGS-mediated PhNF-YC2-silenced plants..pdf]

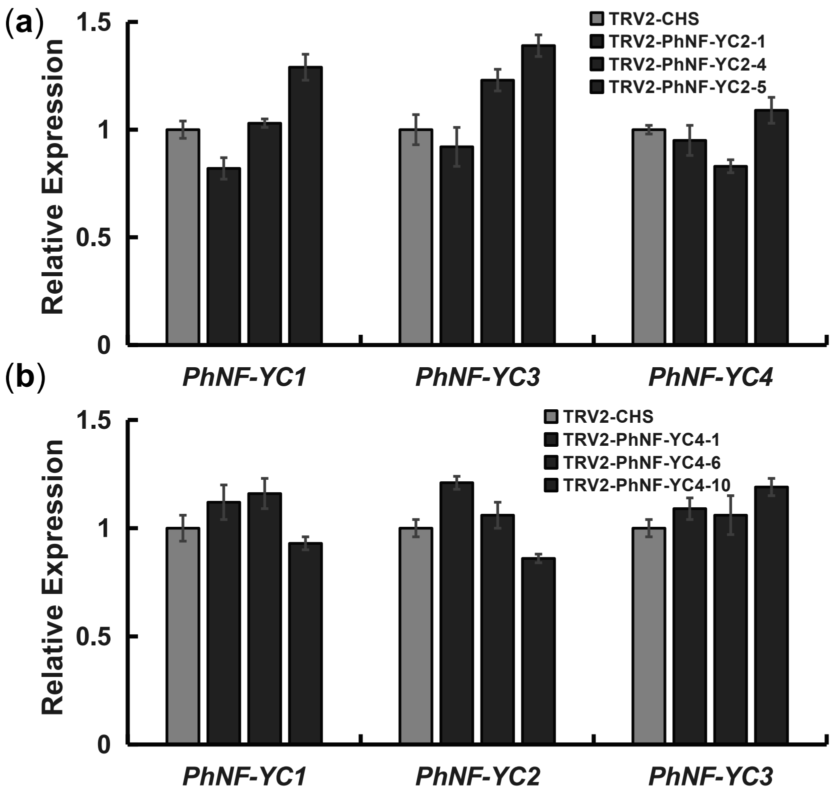

Figure S1. Relative expression levels of other PhNF-YCs in PhNF-YC2-silenced plants (**a**) and PhNF-YC4-silenced plants (**b**) .
